# Long-term performance of right ventricular pacing leads: risk factors associated with permanent right ventricular pacing threshold increase

**DOI:** 10.1007/s10840-018-0481-5

**Published:** 2018-11-06

**Authors:** Hui Peng, Zhijun Sun, Heping Zhang, Wenying Ma

**Affiliations:** Section of Electrophysiology, Division of Cardiology, Beijing Friendship Hospital Affiliated to Capital Medical University, No. 95 Yongan Road, Xi-cheng District, Beijing, 100050 China

**Keywords:** Cardiac pacemaker, Right ventricular pacing threshold, Diabetes mellitus, Myocardial infarction

## Abstract

**Purpose:**

Right ventricular pacing threshold (RVPT) may rise over time accompanied by the increased use of implantable cardiac pacemakers. However, risk factors for permanent RVPT increase are not fully clarified in patients without definite lead fracture and dislodgment. We aimed to evaluate the long-term performance of RV pacing leads and identify risk factors associated with the occurrence of permanent RVPT increase in this population.

**Methods:**

Patients with first implantation of cardiac pacemakers from January 2008 to June 2016 were consecutively enrolled. Follow-up for RVPT increase was until December 2017. The clinical data, specific data on the pacemaker implantation, and routine follow-up were retrieved.

**Results:**

During a follow-up duration of 5.4 ± 2.1 years, permanent RVPT increase (except lead fracture and dislodgment) was found in 8.4% (87/1033) patients. Patients with permanent RVPT increase had higher prevalence of myocardial infarction (MI), diabetes, and the use of amiodarone. The risk factors independently associated with permanent RVPT increase were MI (HR = 1.094, 95% CI 1.014–1.180, *p* = 0.031), diabetes (HR = 2.804, 95% CI 1.064–3.775, *p* = 0.003). MI patients with RVPT increase had higher prevalence of multivessel disease and atrioventricular block. Diabetic patients with RVPT increase exhibited higher serum fasting blood glucose (FBG) and hemoglobin A1c (HbA1c) levels, which were correlated with the maximum RVPT (*p* < 0.001).

**Conclusions:**

Our data showed that permanent RVPT increases (except lead fracture and dislodgement) during long-term follow-up after pacemaker implantation. The likely risk factors predisposing to chronic permanent RVPT increase are MI and diabetes with higher FBG and HbA1c levels.

## Introduction

With the aging population and improved quality of life, the implantation rate of cardiac pacemaker (PM) is increasing. Although there have been technological enhancements in pacing lead manufacturing [1], right ventricular (RV) pacing threshold (PT) may rise over time, shortening the battery life of PM and posing a safety issue [2, 3]. The prevalence of RVPT increase varies from 4 to 25% depending on the type of study [3–6]. As the PM patient population expands in size and longevity, the issue of permanent RVPT increase is likely to become more significant.

The presence of RVPT increase on routine PM’s interrogation may elicit an evaluation for underlying reasons or risk factors. Some different possible risk factors, such as myocardial infarction (MI), hyperkalemia, or antiarrhythmic drugs associated with a higher incidence of RVPT increase, have been reported [7–9]. But systematic data on permanent RVPT increase after PM implantation are scarce, and data on pre-operative, peri-procedural, and post-operative characteristics associated with RVPT increase have not been fully clarified, especially in patients without definite lead fracture and dislodgement. The purpose of the present study is to characterize the chronic performance of RV pacing leads during long-term follow-up after PM implantation and to identify clinical risk factors associated with permanent RVPT increase in this population.

## Patients and methods

### Study population

**Fig. 1:**
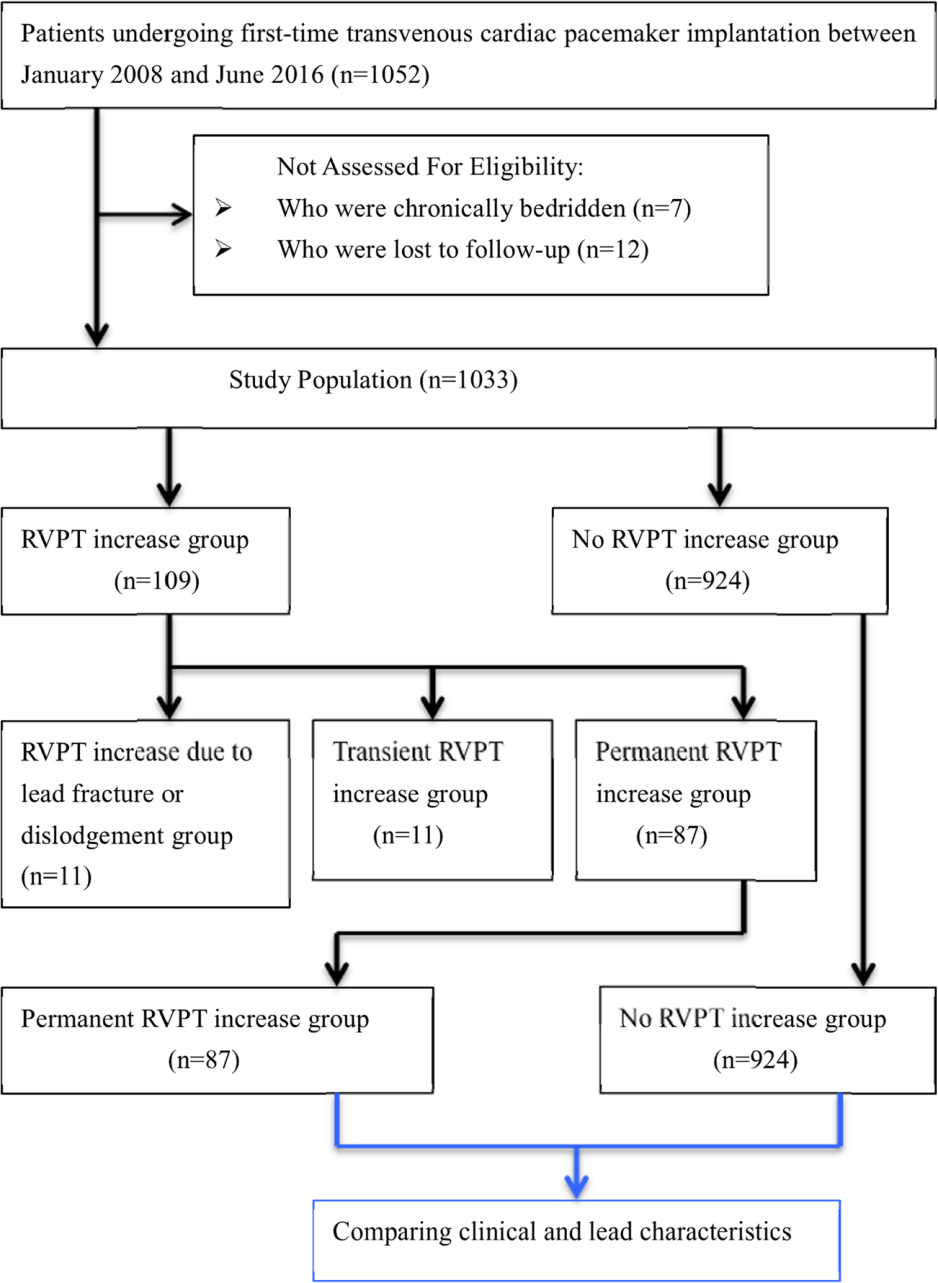
Patient selection flow. After excluding patients who were chronically bedridden (*n* = 7) and who were lost to follow-up (*n* = 12), we ultimately enrolled 1033 eligible patients

We retrospectively enrolled a total of 1033 patients who underwent first-time transvenous PM implantation from January 2008 to June 2016 at our institution and had a long-term follow-up visit. We first identified 1052 consecutive patients who received a PM during this period. We excluded patients who were chronically bedridden (*n* = 7) or who were lost to follow-up (*n* = 12). After exclusion, we ultimately enrolled 1033 eligible patients. The patients were divided into two groups for analysis: those who developed permanent RVPT increase (except lead fracture and dislodgement) and those who did not (Fig. 1). The implantation indications included symptomatic bradycardia without a reversible etiology.

### Device and lead implantation procedure

All patients had undergone conventional transvenous PM implantation. The PM system manufacturers were Medtronic (MDT; Minneapolis, MN, USA). These PMs included automated atrial and ventricular threshold capture management and enhanced pacing management algorithms (EnPulse, Sensia, Adapta, Medtronic, MN, USA). Each patient received Medtronic 4074/5076 or Vitatron IMK49B/ICQ09B as RV pacing lead. Ventricular leads were passive (Medtronic 4074 and Vitatron IMK49B) or active fixation (Medtronic 5076 and Vitatron ICQ09B) mechanism and were implanted into the RV apex or interventricular septum via a percutaneous subclavian vein puncture. The atrial lead was predominately placed at the right atrial appendage. Lead and device testing (pacing and sensing thresholds, pacing impedance) was performed at a pulse width of 0.4 ms. Individual written informed consent for the PM implantation procedure was obtained from all patients.

### Patient follow-up and data collection

All patients were followed in our PM clinic. Patients were seen 2 weeks after device implantation and 1, 3, 6, and 12 months thereafter and then every 6 months until December 2017, performing standard follow-up by the collection of measured data (pacing and sensing thresholds, pacing impedance, and battery status) with special focus on the RVPT evaluated at 0.4-ms impulse width. Electrical data were collected in both bipolar and unipolar modes. Lead integrity was always ensured upon detection of a threshold increase and changes of pacing impedance compared to the previous follow-up. Real-time telemetry measurements were taken using a Medtronic Model 5831 programmer.

Clinical data were extracted from the medical records and included demographics, the indication for PM implantation, underlying chronic medical comorbidities, as well as medical therapy. Follow-up data were acquired up to December 2017. During follow-up, venous blood was collected every 12 months for all patients. The serum potassium, creatinine, and superoxide dismutase (SOD) were measured enzymatically. Quantification of malondialdehyde (MDA) and hemoglobin A1c (HbA1c) was done using a high-performance liquid chromatography method. Fasting blood glucose (FBG) was measured by a glucose oxidase procedure. Left ventricular ejection fraction (LVEF) was measured by echocardiography, using Simpson’s biplane method. The diastolic function was classified from measurements of color M-mode.

### Definition and reaction to an RVPT increase

PT and pulse width are defined as the minimum amount of energy needed to capture the myocardial tissue electrically. Based on previous observations [4, 10, 11], the day-to-day fluctuations reported being ≤ actual PT + 1 V. According to our mean RVPT at implantation (0.47 ± 0.13 V@0.4 ms), we defined a permanent RVPT increase as PT being constantly ≥ 1.5 V at 0.4 ms over 6 months. The onset of a permanent RVPT increase ≥ 1.5 V was traced back according to threshold data as stored by PMs.

Once increased RVPT occurred, pulse width and/or output could be increased to improve the safety margin by PM automatic verification of capture. Lead integrity and dislodgement should be examined by lead impedance measurement and X-ray lead inspection. Further follow-up visit was scheduled monthly until the RVPT showed fluctuations ≤ 1 V. Otherwise, we would insert a new RV lead. A high-threshold warning is issued if the amplitude threshold is > 2.5 V; the PM responds by adapting to an amplitude of 5.0 V and a pulse width of 1.0 ms.

### Statistical analysis

The statistical analysis is exploratory. We wanted to evaluate the association of different variables with permanent RVPT increase (except lead fracture and dislodgement). Univariate analysis was used to detect increased RVPT associations. These analyses were performed by: (a) Student’s *t* test, for comparing the normally distributed continuous variables; (b) Pearson’s *χ*^2^ test, for comparing the categorical variables; (c) Pearson’s correlation method, for examining the correlations between the maximum ventricular PT values and the levels of HbA1c, FBG, and oxidative stress markers during follow-up; and (d) multivariable analysis. Multivariable Cox proportional hazard analysis was performed to estimate the risk factors associated with the chronically permanent RVPT increase. All variables with *p* < 0.10 from univariate analysis were entered in the multivariable Cox proportional hazard model. Adjusted hazard ratios (HRs) with their 95% confidence intervals are reported. Continuous variables are expressed as mean ± standard deviation and categorical variables as relative frequencies (percentage). For each biochemical factor, the average parameters per patient during follow-up were calculated and included in the final analysis. A two-sided *p* value of < 0.05 was considered statistically significant. All statistical analyses were performed with the SPSS software version 20.0 (IBM Corp., Armonk, NY, USA).

## Results

### Characteristics of patients and ventricular leads

Of these 1033 patients, all demographic data, cardiovascular risk profile, biochemical results, medical therapy, and electrical data are detailed in Table 1. The Medtronic 4074 or 5076 RV electrodes were implanted in 81% (837/1033) patients. The remaining patients (196/1033, 19%) were implanted with the Vitatron IMK49B/ICQ09B. Model 5076 is a bipolar, co-radial, silicone rubber insulated, and steroid-eluting pacing lead. Model IMK49B/ICQ09B and Model 4074 are all silicone inner/polyurethane outer insulated, steroid-eluting, bipolar leads. In the present study, no deaths occurred as a direct result of PM placement. Overall, in-hospital surgical complications included pneumothorax in 0.48%, pocket haematoma in 0.77%, haemothorax in 0.19%, and device infection in 0.09%. Lead fracture and dislodgement occurred in 1.0% of all implantations.

**Table 1:** General characteristics of the study population

Variable	Study population *n* = 1033	Permanent RVPT	Increase^a^	*p* value^b^
		No (*n* = 924)	Yes (*n* = 87)	
Age (at implantation), year	68.54 ± 9.08	68.47 ± 9.06	68.98 ± 8.68	0.609
Male sex, *n* (%)	561(54.3)	494(53.4)	47(54.0)	0.920
Pacing indications, *n* (%)				
Sick sinus syndrome	492(47.6)	444(48.0)	40(46.0)	0.711
AV block	363(35.1)	326(35.3)	30(34.5)	0.881
AF with bradycardia	178(17.3)	154(16.7)	17(19.5)	0.494
Medical records, *n* (%)				
CAD	481(46.5)	419(45.3)	48(55.2)	0.079
myocardial infarction	167(16.2)	142(15.4)	21(24.1)	0.033*
Hypertension	536(51.9)	471(51.0)	45(51.7)	0.894
Diabetes	409(39.6)	350(37.9)	44(50.6)	0.020*
LVEF, %	55.76 ± 5.76	55.83 ± 5.79	55.06 ± 5.36	0.241
Serum creatinine, μmol/l	85.75 ± 17.12	85.46 ± 17.30	89.60 ± 14.19	0.126
Serum potassium, mmol/l	4.14 ± 0.20	4.13 ± 0.20	4.16 ± 0.23	0.294
Medications, *n* (%)				
ACE inhibitor or ARB	484(46.9)	429(46.4)	42(48.3)	0.741
β-blocker	293(28.4)	259(28.0)	24(27.5)	0.930
Aldosterone antagonist	153(14.8)	132(14.3)	14(16.1)	0.647
Statins	525(50.8)	457(49.5)	48(55.2)	0.308
Amiodarone	160(15.5)	137(14.8)	20(22.9)	0.044^*^
Device related characteristics (at implantation), *n* (%)				
Active fixation	424(41.3)	381(41.2)	37(42.6)	0.815
Apical pacing	655(63.4)	595(64.4)	55(63.2)	0.827
Dual-chamber systems	835(80.8)	742(80.3)	71(81.6)	0.769
RVPT (at 0.4 ms), V	0.47 ± 0.13	0.46 ± 0.12	0.45 ± 0.13	0.481
RV sensing, mV	12.12 ± 3.24	12.13 ± 3.20	12.93 ± 3.13	0.158
RV impedance, Ω	654.8 ± 108.6	659.4 ± 108.7	640.3 ± 107.5	0.328

AF, atrial fibrillation; CAD, coronary artery disease; LVEF, left ventricular ejection fraction; ACE, angiotensin-converting enzyme; ARB, angiotensin receptor blocker; RVPT, right ventricular pacing threshold

^a^Excluding lead fracture and dislodgement

^b^Comparison of general characteristics of patients with and without permanent RVPT increase

*Significant

### Changes in RVPT during follow-up

#### Patients with increased RVPT

**Fig. 2:**
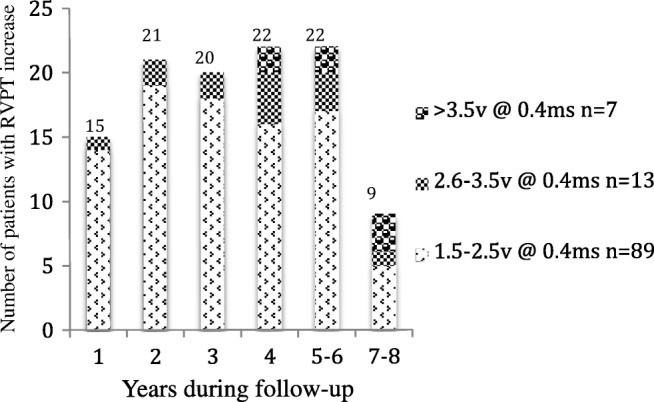
Patients with right ventricular pacing threshold (RVPT) increase during follow-up. Onset of RVPT increase was traced back by the devices. Patients were allocated according to the time of their maximum RVPT reached. The maximum RVPT increase occurred within the first year from implantation in 13.8% (15/109) patients, within the second year in 19.3% (21/109) patients, between the third and the sixth year in 58.7% (64/109) patients, and after the sixth year in 8.2% (9/109) patients

During the long-term follow-up (mean duration 5.4 ± 2.1 years), RVPT increase was found in 10.5% (109/1033) patients. RVPT was between 1.5 and 2.5 V in 8.6% (89/1033) patients, whereas it was between 2.6 and 3.5 V in 1.2% (13/1033) patients, above 3.5 V in 0.7% (7/1033) patients. These patients can be classified into two groups according to the evolution of ventricular PT: group 1 (*n* = 11): transient RVPT increase and group 2 (*n* = 98): permanent RVPT increase during follow-up (Fig. 1). The timing of RVPT increase was different (Fig. 2).

In the group 2, except lead fracture and dislodgement (1.0%, 11/1033), 8.4% (87/1033) patients had chronically permanent RVPT increase for a subsequent evaluation and analysis (Fig. 1). In those patients, the impedance did not change significantly during follow-up, and the variation of R-wave amplitude throughout the study ranged from 5 to 20 mV; no oversensing or undersensing was seen. No patients had symptoms in the event of an RVPT increase because of automatic reversion into the safe pacing setting. No adverse event was reported at one-month follow-up visit after reprogramming, nor at the ensuing follow-up visits.

#### The risk factors associated with chronically permanent RVPT increase

There were 924 patients in the no RVPT increase group and 87 patients in the permanent RVPT increase group. We compared the clinical and lead characteristics between these two groups (Table 1). Among the clinical differences observed, MI (including a history of MI and newly diagnosed MI during follow-up) (24.1% vs 15.4%, *p* = 0.033), diabetes (50.6% vs 37.9%, *p* = 0.020), and the use of amiodarone (22.9% vs 14.8%, *p* = 0.044) were statistically seen more frequently in the permanent RVPT increase group. Although there was a trend toward more patients with coronary artery disease among the RVPT increase group, the difference did not reach statistical significance. No other clinical variables were found to be significantly different. Additionally, no significant differences were found concerning the general parameters of lead electrical performance, fixation mode, pacing position, and single or dual chamber system. There was no difference in RVPT increase among these lead types irrespective of passive or active fixation mode. Upon multivariable Cox proportional hazard analysis, MI (including a history of MI and newly diagnosed MI during follow-up) (HR = 1.094, 95% CI 1.014–1.180, *p* = 0.031) and diabetes (HR = 2.804, 95% CI 1.064–3.775, *p* = 0.003) appeared to be significantly associated with chronically permanent RVPT increase, after adjusting for possible confounders (Table 2).

**Table 2:** Multivariate cox model with the risk of chronically permanent RVPT increase (excluding lead fracture and dislodgement)

Variable	Hazard ratio	95% confidence interval	*p* value
Coronary artery disease	0.524	0.341–1.310	0.269
Diabetes	2.804	1.064–3.775	0.003^*^
Myocardial infarction	1.094	1.014–1.180	0.031^*^
Amiodarone	0.732	0.366-1.322	0.158

*Significant

In our MI population, 153 patients were revascularized (94.3% in RVPT increase group vs 90.5% in no RVPT increase group, *p* > 0.05). MI patients with RVPT increase exhibited a higher rate of multivessel disease (61.9% vs 37.9%; *p* = 0.037), but they were less likely to receive reperfusion therapy acutely (57.1% vs 82.8%; *p* = 0.007) than those without RVPT increase. Newly diagnosed acute MI was present in 61% (100/163) MI patients during follow-up, and 16 patients of those newly diagnosed MI were in the RVPT increase group. There was a significantly higher prevalence of atrioventricular block (PM rhythm) (52.4% vs 18.3%; *p* = 0.001) in MI patients with RVPT increase. Of these PM-dependent MI patients with RVPT increase, ten patients were diagnosed as NSTEMI initially, and 90% of those initial categorizations of NSTEMI were changed in diagnosis toward STEMI during hospitalization.

**Fig. 3:**
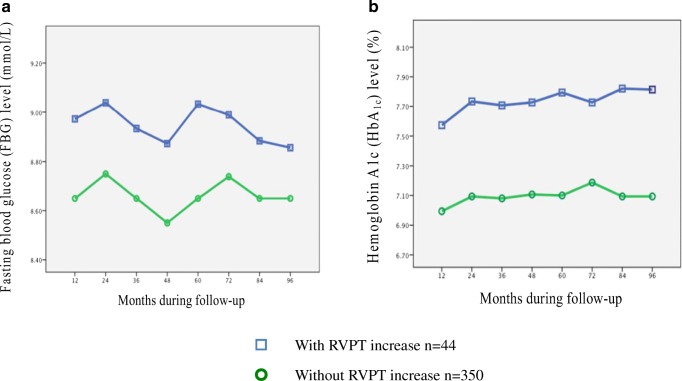
Changes in serum fasting blood glucose (FBG) levels (**a**) and changes in serum hemoglobin A1c (HbA1c) levels (**b**) at periodic intervals during follow-up, comparing diabetic patients with and without permanent right ventricular pacing threshold (RVPT) increase. The differences were significant at every time point between two groups (*p* < 0.05). The mean serum FBG level was higher in diabetic patients with permanent RVPT increase (9.39 ± 0.75 vs 7.70 ± 0.79, *p* = 0.001). The mean serum HbA1c level was higher in diabetic patients with permanent RVPT increase (8.01 ± 0.52 vs 6.82 ± 0.44, *p* = 0.001)

**Fig. 4:**
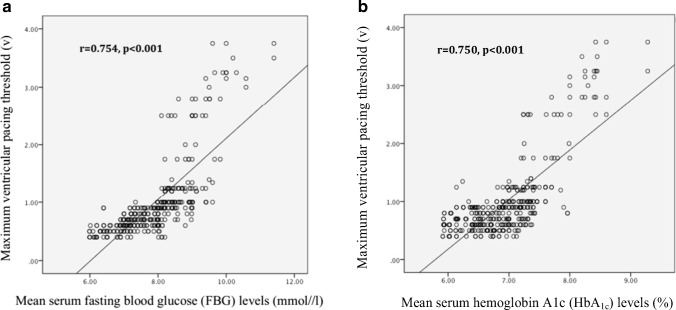
**a** Correlation between maximum ventricular pacing threshold values and mean serum fasting blood glucose (FBG) levels during follow-up in the diabetic patients. **b** Correlation between maximum ventricular pacing threshold values and mean serum hemoglobin A1c (HbA1c) levels during follow-up in the diabetic patients

In our diabetic population, the mean duration of diabetes was 8.1 ± 3.3 years. Newly diagnosed diabetes was present in 18% patients during follow-up. All patients used oral hypoglycemic medications, whereas 20.2% patients received insulin. Diabetic patients with permanent RVPT increase had significantly higher FBG and HbA1c at periodic intervals during follow-up (Fig. 3). According to linear correlation analysis, serum FBG and HbA1c levels were found to be positively associated with the maximum RVPT in diabetic patients during follow-up (*p* < 0.001) (Fig. 4). Notably, although there was a trend toward a lower LVEF (53.34 ± 5.26 vs 55.01 ± 6.17, *p* = 0.088) and a higher mitral E/E’ ratio (7.70 ± 1.25 vs 7.31 ± 1.37, *p* = 0.070) in RVPT increase group, no significant differences were found compared to diabetic patients without RVPT increase. Additionally, presence of other concomitant diseases and the parameters of lead electrical performance were similar between the two subgroups (*p* > 0.05). Meanwhile, we analyzed and compared the serum SOD and MDA levels. Compared with diabetic patients without RVPT increase, the mean serum SOD level was lower in diabetic patients with permanent RVPT increase (46.32 ± 5.17 u/ml vs 60.64 ± 3.61 u/ml, *p* < 0.001), while mean serum MDA level was higher (10.37 ± 0.71 nmol/ml vs 7.83 ± 1.42 nmol/ml, *p* < 0.001), which suggested the increased level of oxidative stress. According to linear correlation analysis, serum SOD and MDA levels were inversely and positively associated with the maximum RVPT, respectively, in diabetic patients during follow-up (SOD: *r* = 0.872, *p* < 0.001; MDA: *r* = 0.734, *p* < 0.001).

## Discussion

Accompanied by the increased use of implantable cardiac PM, one of the issues is the RVPT increase over a long-term follow-up. In this retrospective analysis, we characterized the chronic behavior of RV leads and found that about 10.5% of patients had an RVPT increase. Moreover, 2.0% of patients had an increased threshold > 2.5 V, which requires a high current drain for pacing. With regard to the RVPT increase, interestingly, we noticed that risk factors associated with chronically permanent RVPT increase (except lead fracture and dislodgement) might be diabetes and MI, which might influence the excitability of the cardiac tissue and the extent of local tissue fibrosis.

### Characterization of the RVPT increase

Previous studies had shown that standard steroid-eluting pacing leads efficiently reduced the PT increase caused by inflammatory reactions at the tissue-lead interface after implantation [12]. This effect was stable and maintained during 5- or 10-year follow-up [13, 14]. Nonetheless, Biffi et al. [4] reported a permanent RVPT increase > 3 V at 0.4 ms in 4 of 126 patients (3%) beyond 1 year in a single-center experience. Kistler [5] and Medi [6] reported 4% patients and 2% patients with increased RVPT (> 2 V at 0.4 ms) at a 24-month follow-up and 6-month follow-up, respectively. These short-term observational studies along with our long-term follow-up findings supported that RVPT might increase in patients with modern leads, and a relevant permanent RVPT increase ≥ 2.5 V at 0.4 ms occurred in about 2–4% of patients. Moreover, it was similar frequency of PT increase in first 6 years of follow-up, which was suggestive that RVPT increase might happen at any time during follow-up, and patients might be vulnerable at this event at any time.

### The clinical characteristics of patients with chronically permanent RVPT increase

The RVPT increase likely reflects the complex nature of the patient population with multiple diseases and physiological or pharmacological conditions.

Cui et al. [15] demonstrated that acute ischemia for more than 30 min could increase PT almost threefold to the baseline level. Local ischemia over several hours can change myocardial resistivity dramatically. The electrical uncoupling of myocardial cells by ischemia leads to an increase in intracellular resistance of 50–100% [16]. Recently, Chen et al. [17] and Upadhyay et al. [18] reported that PM function returned back to normal after stent implantation. Pivatto et al. [19] described improvement of PT following early reperfusion recently. But in the present study, we found MI remained associated with chronically permanent RVPT increase in spite of the fact that 94% MI patients were revascularized. This effect could be explained by increased myocardial ischaemia with multivessel lesions in patients with RVPT increase. Also, it could be due to the diagnostic challenges of acute MI in PM-dependent patients because of atrioventricular block. We observed that 52.4% of MI patients with RVPT increase had PM rhythm. Of those patients, more initial categorizations of NSTEMI were changed in diagnosis toward STEMI during hospitalization. As the initial electrocardiogram in acute MI patients is very important for the acutely invasive therapy, it is not surprising that patients with paced rhythm are less likely to receive primary angioplasty compared to patients without paced rhythm. Further research into this question seems to be warranted.

On the other hand, the association of chronically permanent RVPT increase with diabetes is very interesting. In the present study, according to diabetic subgroup analysis, the patients with permanent RVPT increase had higher FBG and HbA1c than those without RVPT increase. Furthermore, the circulating FBG and HbA1c were correlated with the maximum RV threshold in the diabetic patients. Stern et al. [20] reported that diabetes affected electrical activity of the heart. Zdarska et al. [21] also revealed the subtle changes of the electrical heart field by body surface electrocardiogram maps and significantly decreased amplitudes of R waves in diabetic patients. These studies along with our findings suggested that hyperglycemia might influence the excitability of the cardiac tissue.

Further analysis revealed that the diabetic patients with permanent increased RVPT exhibited increased oxidative stress. The circulating MDA and SOD levels were correlated with the maximum RV threshold in the diabetic patients. Aging and death of human myocardial cells mediated by the activation of oxidative stress result in accumulating deposition of interstitial fibrotic tissue and changes in myofibrillar proteins, which cause myocardial fibrosis and degeneration [22, 23]. Similarly, we noticed that the diabetic patients with RVPT increase tended to have lower LVEF and higher mitral E/E’ ratio. Although no statistical differences were found, those findings might be in accordance with the hypotheses of increased myocardial stiffness, increased resting myocyte tension and myocardial fibrosis associated with diabetic cardiomyopathy. These effects of hyperglycemia on cardiomyopathic mechanisms might theoretically influence the threshold of myocardial excitability, rendering the diabetic myocardium more prone to adversely affect the RVPT.

We also observed a more use of amiodarone in the RVPT increase group. Although the multivariable analysis did not reach statistical difference, this issue would be clinically important because permanent pacing is often used concomitantly with antiarrhythmic drugs. Some reports found an association between chronic amiodarone therapy and a significant rise in the defibrillation threshold [9, 24]. But Huang et al. [25] studied the influence of different antiarrhythmic drugs including amiodarone on the chronic PT in healthy dogs. They found that those drugs did not affect the chronic PT nor the endocardial R wave amplitude. In the present study, amiodarone was prescribed for the medical conditions, such as ventricular arrhythmia or atrial fibrillation. Most patients who used amiodarone had underlying cardiac disease. It was possible that these patients had an intrinsically higher risk of developing RVPT increase. Therefore, we speculated these data could possibly be explained by a different effect of amiodarone in the presence or absence of an underlying disease.

## Implications

In a clinical context, the subclinical impairments of permanent RVPT increase are recognized to affect the longevity of the pacing system, even carry a safety issue. Therefore, the results from the present study further emphasize that patients with associated risk factors for permanent RVPT increase (except lead fracture and dislodgement), such as MI, diabetes with higher FBG and HbA1c, should be considered especially risky patients and taken into account in treatment and care.

## Limitations

This was a retrospective, single-center, observational study, and the results of the analysis must be interpreted in light of this; however, this type of study reflects habitual clinical practice. Another limitation was the small study population. Although there was a significant tendency toward increased RVPT in MI patients and diabetic patients with higher FBG and HbA1c, larger studies with longer follow-up are needed to further delineate the difference and possible risk factors on permanent RVPT increase. Finally, our findings are limited to the types from a single manufacturer (Medtronic), and cannot be extrapolated to all PM leads.

## Conclusions

Our data showed that permanent RVPT increases (except lead fracture and dislodgement) during long-term follow-up after PM implantation. The likely risk factors predisposing to chronic permanent RVPT increase are MI and diabetes with higher FBG and HbA1c levels. Further studies addressing the long-term lead behavior in larger population are warranted.
